# Primary Murine Macrophages as a Tool for Virulence Factor Discovery in Coxiella burnetii

**DOI:** 10.1128/spectrum.02484-21

**Published:** 2022-08-01

**Authors:** Elizabeth Di Russo Case, Saugata Mahapatra, Caitlyn T. Hoffpauir, Kranti Konganti, Andrew E. Hillhouse, James E. Samuel, Erin J. Van Schaik

**Affiliations:** a Department of Veterinary Sciences, University of Wyoming, Laramie, Wyoming, USA; b Department of Microbial Pathogenesis and Immunology, Texas A&M University Health Science Center, Bryan, Texas, USA; c Institute for Genome Sciences and Society, Texas A&M University, College Station, Texas, USA; University of North Dakota

**Keywords:** *Coxiella*, primary macrophages, type IVB secretion, phenotypic profiling, innate immunity

## Abstract

Coxiella burnetii requires a type IVB secretion system (T4SS) to promote intracellular replication and virulence. We hypothesized that *Coxiella* employs its T4SS to secrete effectors that enable stealthy colonization of immune cells. To address this, we used RNA sequencing to compare the transcriptional response of murine bone marrow-derived macrophages (BMDM) infected with those of wild-type *Coxiella* and a T4SS-null mutant at 8 and 24 h postinfection. We found a T4SS-independent upregulation of proinflammatory transcripts which was consistent with a proinflammatory polarization phenotype. Despite this, infected BMDM failed to completely polarize, as evidenced by modest surface expression of CD38 and CD11c, nitrate production, and reduced proinflammatory cytokine and chemokine secretion compared to positive controls. As these BMDM permitted replication of C. burnetii, we employed them to identify T4SS effectors that are essential in the specific cellular context of a primary macrophage. We found five *Himar1* transposon mutants in T4SS effectors that had a replication defect in BMDM but not J774A.1 cells. The mutants were also attenuated in a SCID mouse model of infection. Among these candidate virulence factors, we found that CBU1639 contributed to the inhibition of macrophage proinflammatory responses to *Coxiella* infection. These data demonstrate that while T4SS is dispensable for the stealthy invasion of primary macrophages, *Coxiella* has evolved multiple T4SS effectors that specifically target macrophage function to proliferate within that specific cellular context.

**IMPORTANCE** Coxiella burnetii, the causative agent of Q fever, preferentially infects macrophages of the respiratory tract when causing human disease. This work describes how primary macrophages respond to C. burnetii at the earliest stages of infection, before bacterial replication. We found that while infected macrophages increase expression of proinflammatory genes after bacterial entry, they fail to activate the accompanying antibacterial functions that might ultimately control the infection. This disconnect between initial response and downstream function was not mediated by the bacterium’s type IVB secretion system, suggesting that *Coxiella* has other virulence factors that dampen host responses early in the infection process. Nevertheless, we were able to identify several type IVB secreted effectors that were specifically required for survival in macrophages and mice. This work is the first to identify type IVB secretion effectors that are specifically required for infection and replication within primary macrophages.

## INTRODUCTION

Coxiella burnetii, the causative agent of Q fever, is a stealth pathogen for which the principal pathogenic niche in humans is the alveolar macrophage (AM) (1). *Coxiella* is primarily transmitted to humans via contaminated aerosols generated where infected sheep, goats, or cattle are housed at high density (2). Q fever has a very low infectious dose, as fewer than 10 organisms can establish disease (3). Q fever usually manifests as a severe acute respiratory illness that may progress to pneumonia or hepatitis. Rarely, Q fever can become a chronic, life-threatening disease. The pathogen C. burnetii is a naturally obligate, intracellular, Gram-negative bacterium that can persist in the environment for extended periods and is resistant to most disinfection methods (4). For these reasons, ease of aerosolization, low infectious dose, and high degree of environmental stability, C. burnetii is categorized as a select agent (SA) in the United States. As such, working with all strains of C. burnetii, except the exempted Nine Mile II (NMII) strain, requires SA registration and biosafety level 3 (BSL3) containment. The attenuated NMII strain (RSA439, phase II, clone 4) was derived from the virulent Nine Mile isolate, has a truncated lipopolysaccharide (LPS), and is avirulent to humans (2, 5). Limited access to BSL3 laboratory space makes working with the NMII strain an attractive model for C. burnetii infection, and most *in vitro* studies of *Coxiella* pathogenesis are conducted with this strain.

As C. burnetii preferentially infects and replicates within macrophages, researchers have attempted to employ primary macrophages in cell culture to model pathogenesis *in vitro*. However, several studies have shown that the attenuated NMII strain of C. burnetii is restricted from colonizing murine bone marrow-derived macrophages (BMDM) (6–8). The bacteria appear to be capable of surviving the entry process and inducing expansion of the *Coxiella*-containing vacuole (CCV). However, replication in these cells has been shown to be highly limited compared to that in permissive cell types, such as stable fibroblast cell lines (8). The restriction of NMII by BMDM had been largely attributed to its truncated O antigen, which likely leaves surface lipoproteins exposed to activate Toll-like receptor 2 (TLR2)-dependent antibacterial defenses (7). Cockrell et al. published a differentiation protocol that employed recombinant macrophage colony-stimulating factor (mCSF) for generating BMDM that were equally permissive for NMII replication as stable cell lines (9). That report was particularly important, as it highlighted that O antigen is not the sole virulence factor contributing to *Coxiella*’s ability to evade detection and prevent proinflammatory macrophage responses (9).

Much work with C. burnetii NMII has focused on the contribution of its type IVB secretion system (T4SS) to the manipulation of macrophage host responses. Unlike the O antigen, the C. burnetii T4SS is absolutely required for intracellular replication (10). T4SS-null mutants activate the proinflammatory transcriptional responses of macrophage cell lines within 48 to 72 h of infection (11, 12), and some effectors have been identified as modulators of the host response to infection. The effector IcaA has been shown to inhibit inflammasome activation (13). Most recently, NopA was described as an effector that inhibits proinflammatory transcriptional responses to *Coxiella* as it replicates (12).

In this study, we differentiated primary macrophages from the bone marrow of C57BL/6 mice and characterized their response to infection with NMII prior to the initiation of replication. Our primary aim was to determine whether *Coxiella*’s stealthy colonization of BMDM (9) could be attributed to T4SS-dependent modulation of host cell polarization at early times. In this study, we found that the T4SS was not required at the initial stages of infection to manipulate macrophage responses.

Nonetheless, the T4SS of Coxiella burnetii is a crucial virulence factor for the pathogen, and many laboratories continue to define the functions of individual effectors. For T4SS effectors that have been genetically inactivated, a large proportion had no discernible contribution to *Coxiella* pathogenesis in permissive cell lines (14). We hypothesized that among those apparently nonessential effectors, one or more effectors are required for modulating a primary macrophage-specific function. To this end, we tested whether BMDM permit the replication of C. burnetii NMII mutants lacking effectors that are dispensable for replication in permissive cell lines. Our screen of mutant C. burnetii did suggest that multiple macrophage-specific effectors are required for intracellular replication in BMDM and disease in an animal model of infection. Further, we identified one effector, encoded by *CBU1639*, that contributes to the inhibition of antibacterial responses. Importantly, these data demonstrate the profound influence that our choice of *in vitro* experimental models have on our ability to identify and understand host-pathogen interactions.

## RESULTS

### Replication of C. burnetii NMII in primary murine macrophages.

**FIG 1:**
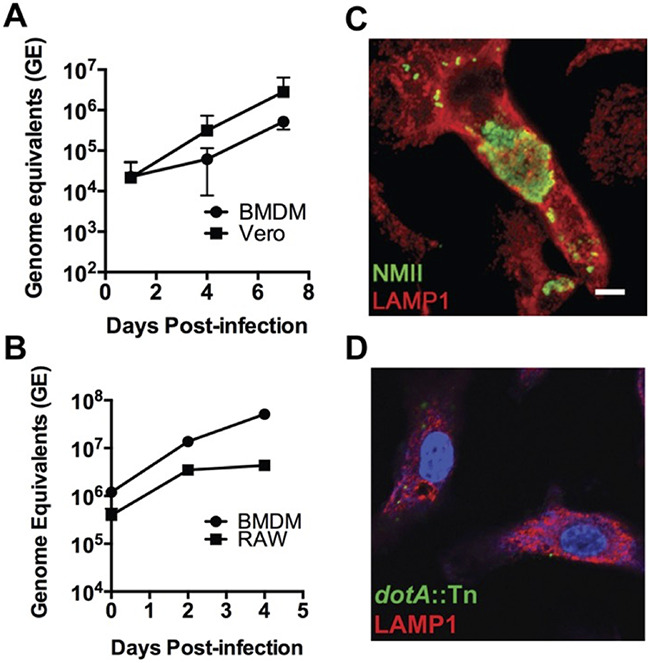
Replication of C. burnetii NMII in murine bone marrow-derived macrophages (BMDM). (A and B) Growth curves of C. burnetii NMII in Vero cells and C57BL/6 BMDM over 7 days (A) or RAW 264.7 (RAW) cells and BMDM over 4 days (B). Each point is the mean of results from 3 independent experiments, with error bars representing standard deviations. (C and D) Confocal micrographs of C. burnetii NMII wild-type-infected (C) or *dotA*::Tn mutant-infected (D) BMDM at 4 days postinfection. *Coxiella* was stained green, LAMP-1 is shown in red, and DNA is stained blue with DAPI. Scale bar, 10 μm.

As our laboratory is interested in modeling the host innate immune response to C. burnetii infection, we sought to develop a primary murine macrophage model of infection. To differentiate murine macrophages from bone marrow, we used L929-conditioned medium as a source of murine mCSF (15). To verify that these BMDM could support NMII replication, infections were conducted in parallel with Vero cells (16) and a mouse macrophage cell line, RAW 264.7 (Fig. 1A and B). We observed that NMII replicated in BMDM with kinetics similar to that of the Vero cells over 7 days. We also observed that NMII replicated better in BMDM (42.5-fold) than in RAW 264.7 cells (11.1-fold) after 4 days of infection. We attributed this difference to the limited life span of RAW 264.7 cells; their rapid proliferation quickly depleted the culture medium. *Coxiella*-containing vacuoles (CCVs) of infected BMDM grow to occupy a substantial proportion of the cell volume and are filled with replicating bacteria by 4 days postinfection (Fig. 1C). As a control, BMDM were also infected with a strain of NMII containing a transposon insertion in the *dotA* gene to demonstrate the requirement for an active type IVB secretion system (T4SS) for intracellular replication of the bacterium. As expected, Fig. 1D shows the lack of CCV expansion and absence of replication for the *dotA*::Tn strain, in direct contrast to the NMII wild type at 4 days postinfection.

### Early transcriptional response of BMDM to Coxiella burnetii NMII.

Having established that these BMDM support replication of C. burnetii NMII, we next examined the host cells’ response to infection with the bacterium. BMDM are known to respond to bacterial infection with rapid upregulation of proinflammatory gene transcription within hours of stimulation (17, 18). Published works characterizing the macrophage transcriptional response to *Coxiella* infection have assessed gene expression after 24 h, with typical time points ranging from 48 to 72 h postinfection (hpi) (11, 12). Given the usually rapid transcriptional kinetics for primary macrophage responses to bacterial infection (17), we wanted to determine how host gene expression might change after the initiation of T4SS effector delivery but before replication is detectable (19). We hypothesized that if there is a T4SS-dependent suppression of host transcription in primary macrophages within the first 8 to 24 h postinfection, we should be able to detect the transcriptional response using the BMDM infection model.

**FIG 2:**
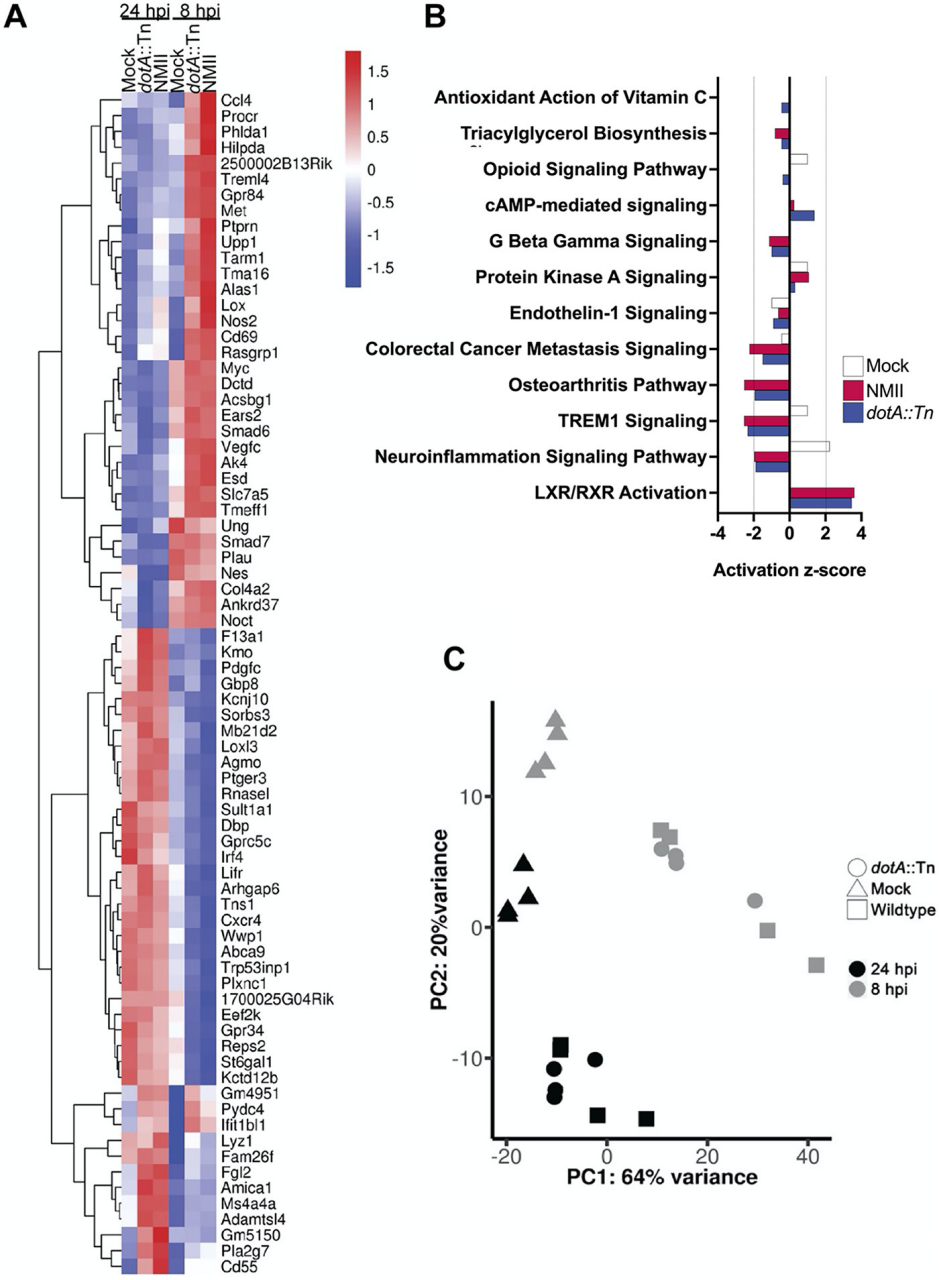
BMDM transcriptional response to C. burnetii at 8 and 24 h postinfection. (A) Heat map displaying the most highly differentially expressed BMDM genes at 8 and 24 h postinfection (hpi). The legend shows the range of log_2_-transformed fold changes of genes either upregulated (red) or downregulated (blue) in response to infection with either wild-type C. burnetii (NMII) or a T4SS-null mutant (*dotA*::Tn). (B) Canonical pathways predicted by Ingenuity Pathway Analysis (IPA) to be differentially regulated in response to infection with wild-type or T4SS-null C. burnetii. (C) Principal-component analysis of the RNA-seq data set. RNA from four independent experiments was sequenced using mock-infected BMDM (triangles) or BMDM infected with either wild-type C. burnetii (NMII, squares) or T4SS-null C. burnetii (*dotA*::Tn, circles) at 8 hpi (gray) and 24 hpi (black).

To address these questions, we infected BMDM with the NMII wild type or a *dotA*::Tn strain and harvested the total host mRNA at 8 and 24 h postinfection and subjected it to transcriptome sequencing (RNA-seq), using uninfected BMDM as a control. In choosing this time range, we minimized the possibility of confounding effects due to *Coxiella* replication, which could not be controlled for with the *dotA*::Tn mutant. We found that for both wild-type NMII and the *dotA*::Tn mutant, most differential gene expression was observed at 8 h postinfection (Fig. 2A). This is the first report to show a significant upregulation of host gene expression in response to C. burnetii so soon after infection. Both C. burnetii strains induced transcription of genes involved in host innate immune responses (*ccl4*, *treml4*, *tarm1*, *nos2*, *cd69*, *rasgrp1*), lipid metabolism (*hilpda*, *alas1*, *gpr84*, *acsbg1*), and nucleotide metabolism (*dctd*, *upp1*, *ak4*) at 8 h postinfection. Wild-type NMII induced higher levels of expression for these genes than the *dotA*::Tn strain (Fig. 2A). Infection with both strains of *Coxiella* also repressed expression of several transcripts at 8 h, including genes with roles in cell adhesion and migration (*sorbs3*, *loxl3*, *arhgap6*, *tens1*, *plxnc1*), lipid metabolism (*agmo*, *st6gal1*), and innate immune signaling (*irf4*, *lifr*, *cxcr4*). As the principal-component analysis shows, we also observed modest transcriptional changes in the uninfected control cell populations between 8 and 24 h (Fig. 2C). We attribute these differences to the continued development of the BMDM that occurs between 7 and 10 days postdifferentiation, which has been previously documented (20–23). As the largest differences in host gene expression were observed during infection with both strains of *Coxiella*, we concluded that at early times, the host transcriptional response to infection is T4SS independent.

To search for larger patterns in the gene expression data set, we employed the Ingenuity Pathway Analysis (IPA) software package available from Qiagen. For this analysis, we compared the expression of transcripts from each condition (mock, wild-type NMII, and *dotA*::Tn) at 24 h to that at 8 h, to detect infection-dependent gene regulation that occurred within that time window. The analysis predicted that several pathways were differentially regulated between 8 and 24 h following C. burnetii infection (Fig. 2B). The largest activation scores returned by the software package were assigned to signaling pathways associated with innate immune responses to infection. IPA gave the TREM1 and neuroinflammation signaling pathways negative activation scores in contrast to that of mock-infected controls. Both signaling pathways are activated in response to engagement of Toll-like receptors upon infection (24, 25). Interestingly, downregulation of *trem1* gene expression has been associated with acute Q fever (26). The pathway analysis also predicted positive activation of LXR/RXR signaling in response to C. burnetii. Like the TREM1 and neuroinflammation pathways, LXR/RXR signaling is also activated by NF-κB downstream of TLRs (27). These predictions suggest that *Coxiella* infection may alter host innate immune responses prior to bacterial replication independently of T4SS.

**FIG 3:**
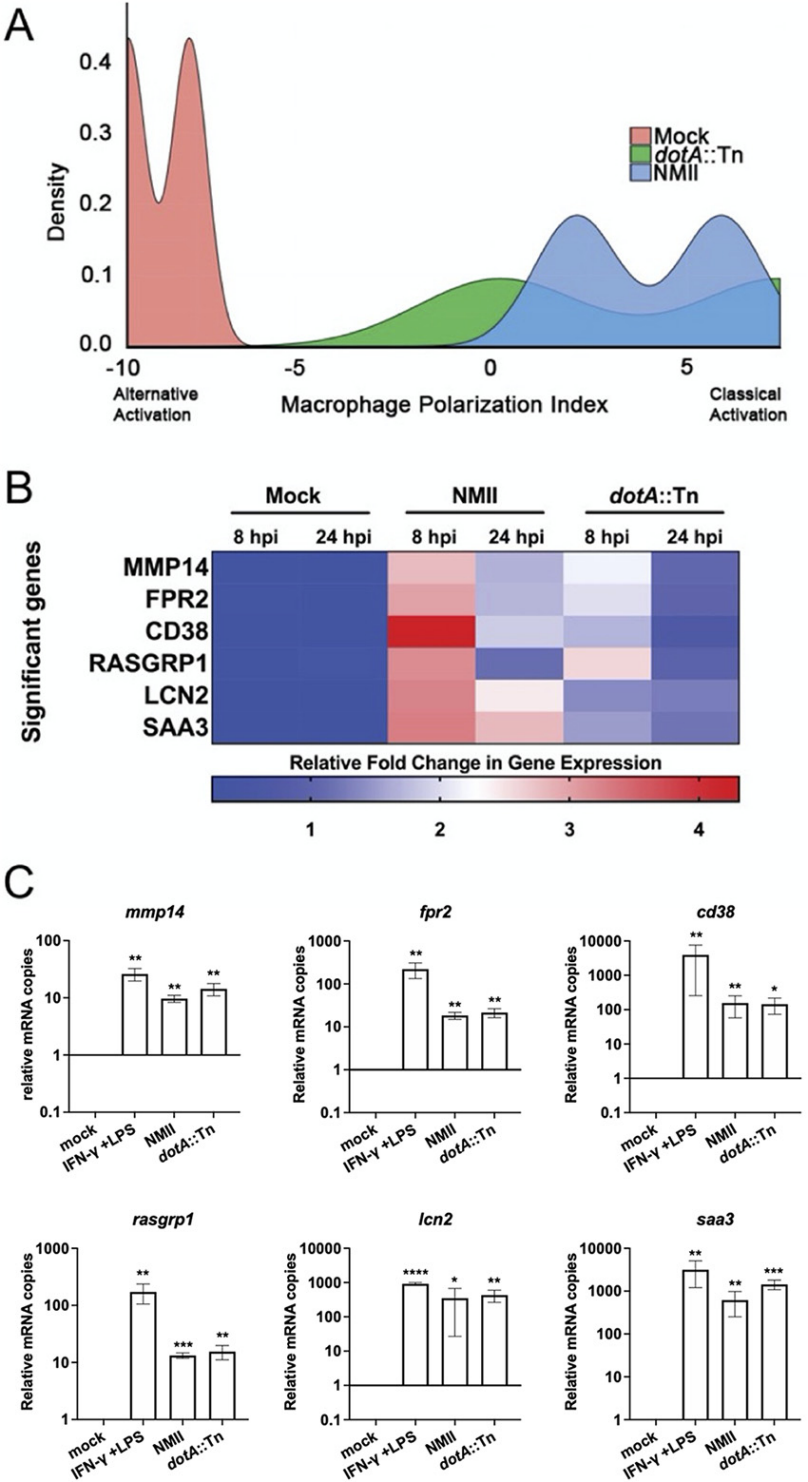
MacSpectrum predicts that C. burnetii infection skews BMDM to a classical activation phenotype. (A) MacSpectrum macrophage polarization index (MPI) graph showing predicted activation status of mock-infected BMDM (pink) or BMDM infected with wild-type NMII (blue) or T4SS-null *dotA*::Tn (green) *Coxiella.* A positive value for MPI indicates a skew toward classical activation, while a negative value indicates alternative activation. (B) Heat map of significant genes returned by MacSpectrum as contributing to the MPI predictions derived from the BMDM RNA-seq data set. The legend shows the range of log_2_-transformed fold changes in gene expression for the given genes. (C) qRT-PCR validation of the significant genes identified by MacSpectrum. Each bar represents the fold change in mRNA copies relative to mock-infected BMDM after normalization to the *actb* transcript. The data presented are the mean of results of three independent experiments with error bars representing standard deviations. Asterisks denote *P* values calculated by a two-tailed ratio-paired *t* test (*, 0.0332; **, 0.0021; ***, 0.0002; ****, <0.0001).

We next subjected the RNA-seq data to analysis with MacSpectrum (28) to predict the polarization phenotypes of the BMDM based on the transcriptional responses we observed at 8 and 24 h postinfection. The MacSpectrum algorithm annotates macrophage populations based upon the relative expression of 500 genes that differentiate classical and alternative macrophage polarization responses (28). This platform grants a negative macrophage polarization index (MPI) to a transcriptional profile that skews toward alternative polarization and a positive MPI to transcriptional profiles consistent with a classical polarization phenotype. Interestingly, for all conditions, a bimodal curve was observed, with the highest absolute value for MPI at 8 h postinfection and then shifting in the opposite direction at 24 h (Fig. 3A). MacSpectrum assigned uninfected BMDM a negative MPI ranging from −9.29 at 8 h to −7.72 at 24 h, indicating that our naive BMDM population skews toward alternative polarization (Fig. 3A) or an anti-inflammatory phenotype. Upon infection with both strains of C. burnetii, BMDM shifted to a positive MPI, as *Coxiella* initially induces a proinflammatory transcriptional program in primary macrophages. We observed a broad distribution of macrophage responses to the *dotA*::Tn strain (7.38 at 8 h, 0.21 at 24 h), whereas wild-type *Coxiella* more consistently induced a classical polarization response (5.89 at 8 h, 2.18 at 24 h) based on the RNA-seq data set. There were 6 signature genes identified by MacSpectrum that most strongly correlated with the predicted polarization state of the BMDM: *mmp14*, *fpr2*, *cd38*, *rasgrp1*, *lcn2*, and *saa3* (Fig. 3B). To validate these data, we used reverse transcription-quantitative PCR (qRT-PCR) to measure transcripts of these significant genes in response to infection or stimulation with an inducer of classical polarization (gamma interferon [IFN-γ] and LPS). At 24 h postinfection, both strains of C. burnetii produced a transcriptional response that was consistent with classical polarization at 24 h (Fig. 3C). For all genes queried, the host transcriptional response to C. burnetii infection was consistent with that for the IFN-γ plus LPS control at 24 h postinfection. These data show that the host transcriptional response to *Coxiella* infection at 24 h is consistent with that of a classically activated macrophage. Notably, the differential gene expression pattern was observed to be independent of the presence of a functional type IVB secretion system.

### Type IVB secretion-independent suppression of the host innate immune response to *Coxiella* infection during initial stages of infection.

**FIG 4:**
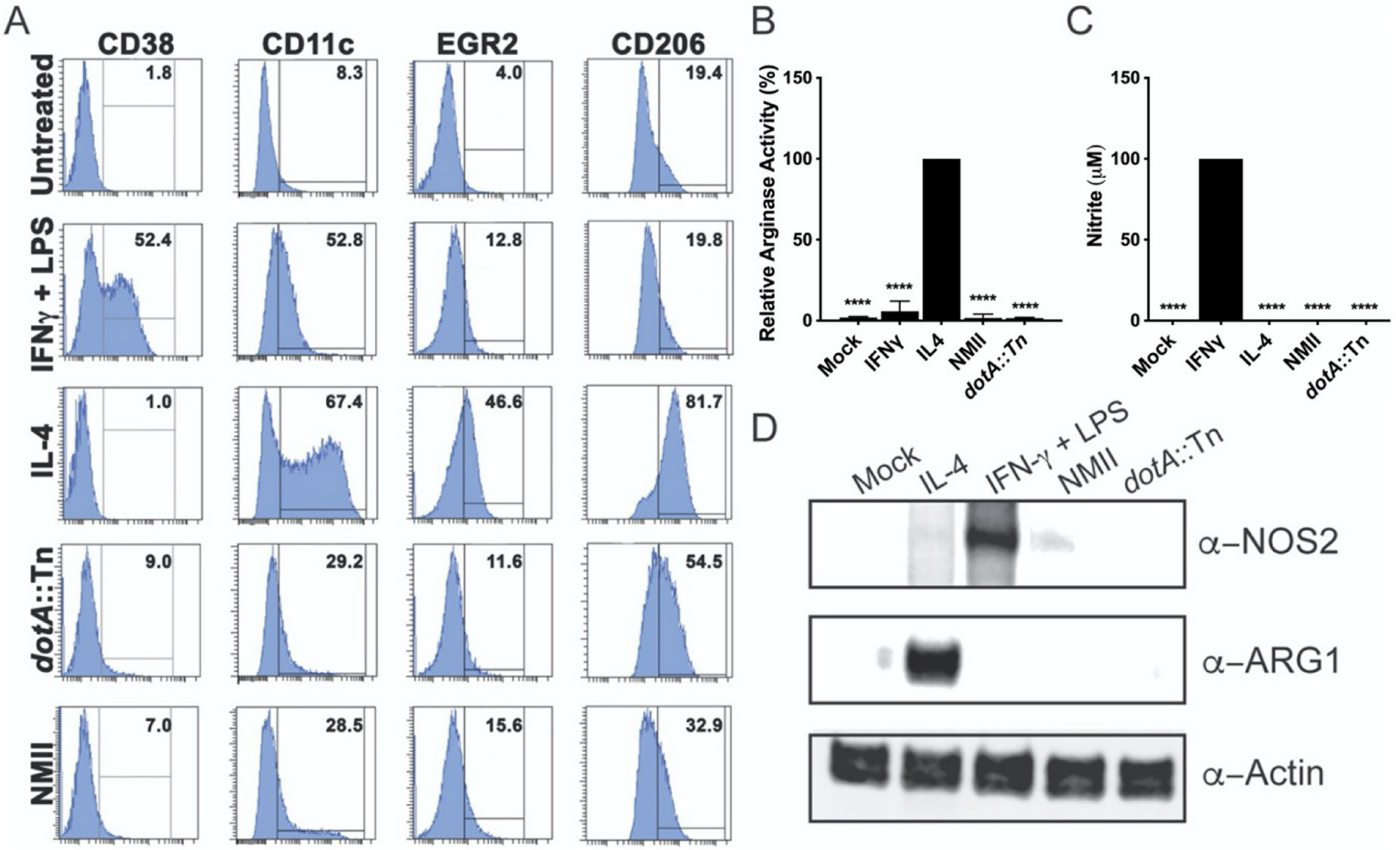
Phenotype of BMDM infected with C. burnetii NMII. (A) Flow cytometric analysis of untreated primary BMDM in parallel with BMDM treated with either IFN-γ plus 100 ng/mL LPS or IL-4 or infected with *dotA*::Tn or wild-type NMII C. burnetii for 48 h. Cells were stained for two markers of classical activation (CD38 and CD11c) and two markers of alternative activation (EGR2 and CD206). A histogram (representative of two independent experiments with greater than 10,000 events) shows the percentage of positive events in the population for each marker and condition, where a horizonal line denotes the threshold for a positive signal. (B) Graph displaying the relative arginase activity of mock-infected BMDM or BMDM treated with either IFN-γ plus 100 ng/mL LPS or IL-4 (positive control, 100%) or infected with *dotA*::Tn or wild-type NMII C. burnetii at 24 hpi. (C) Graph displaying the absolute nitrate production of the same BMDM populations described in the legend for panel B, where the population treated with IFN-γ plus 100 ng/mL LPS serves as the positive control for the assay. For both panels B and C, the data shown are the mean of results of three independent experiments, with error bars representing the standard deviations. The statistical significance relative to positive controls was calculated by one-way ANOVA, with Dunnett’s test for multiple comparisons: ****, *P* < 0.0001. (D) Western blots of whole-cell lysates from mock-infected BMDM, BMDM treated with either IFN-γ plus 100 ng/mL LPS or IL-4, or BMDM infected with wild-type NMII or *dotA*::Tn C. burnetii at 24 hpi. Expression of inducible nitric acid synthase (α-NOS2) and arginase (α-ARG1) is shown, with β-actin (α-actin) serving as a loading control.

We next investigated the polarization phenotype of *Coxiella*-infected BMDM by flow cytometric analysis to determine if the transcriptional responses we observed resulted in the physical expression of a classical activation phenotype. At 48 h postinfection, a time that allowed sufficient stimulation with inducers of alternative (IL-4) and classical (IFN-γ plus LPS) macrophage polarization, we stained infected BMDM for cellular markers of polarization. Uninfected BMDM, which we predicted would adopt an alternative polarization phenotype by MacSpectrum, stained weakly with surface markers EGR2 and CD206, compared with the IL-4-stimulated BMDM, which serve as the control for that phenotype (Fig. 4A). Likewise, they did not display amounts of the classical activation markers CD38 or CD11c comparable to those of the IFN-γ plus LPS control population. For this reason, we define their phenotype as M0, or unpolarized. Indeed, enzymatic assays to measure the activity of arginase and inducible nitric oxide synthase showed that these cells behave as if they are unpolarized (Fig. 4B and C, mock). Surface staining of wild-type NMII- and *dotA*::Tn-infected cells showed upregulation of all surface markers relative to that of uninfected controls, but the percentages of positive cells for CD38, CD11c, EGR2, and CD206 were much lower than that observed for control cells stimulated with IFN-γ plus LPS or IL-4, indicating that the *Coxiella*-induced polarization phenotype is modest. Indeed, despite the significant proinflammatory transcriptional responses we observed (Fig. 3), markers indicative of classical polarization were expressed at approximately 6- to 7-fold (CD38) or 2-fold (CD11c) less than the IFN-γ plus LPS- positive control (Fig. 4A). Moreover, the arginase and nitric oxide synthase activity in these cells was comparable to that of the unstimulated control BMDM (Fig. 4B), suggesting that *Coxiella-*infected BMDM largely behave as unpolarized cells, despite the initial transcriptional responses observed. We confirmed that the lack of enzymatic activity was due to insufficient protein expression (Fig. 4C and D). These results are consistent with our earlier observations for CD38 expression (Fig. 3C); the transcriptional activation we observed for these genes did not result in the production of sufficient protein to mount an effective antibacterial response.

**FIG 5:**
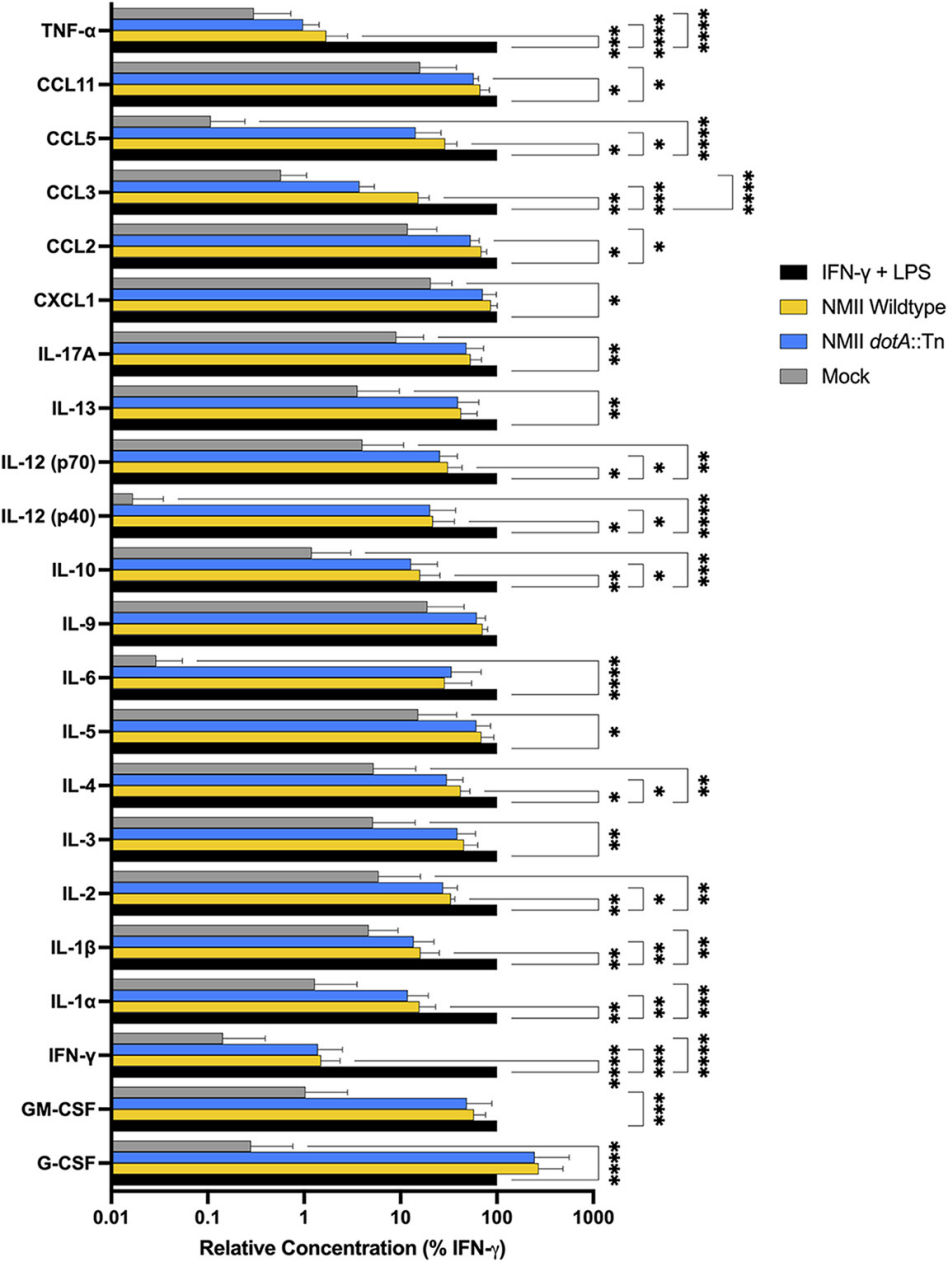
*Coxiella* infection of BMDM induced modest cytokine/chemokine secretion. Bio-Plex cytokine assay demonstrating host cytokine secretion response to mock-infected BMDM (gray bars), BMDM treated with IFN-γ plus 100 ng/mL LPS (black bars), or BMDM infected with wild-type NMII (yellow bars) or *dotA*::Tn (blue bars) C. burnetii at 24 hpi. Each bar represents the mean protein secretion in BMDM supernatants from three independent experiments normalized to the positive control (IFN-γ plus 100 ng/mL LPS). Error bars represent standard deviations from the mean, with asterisks denoting *P* values calculated by two-way ANOVA, with Dunnett’s test for multiple comparisons (*, 0.0332; **, 0.0021; ***, 0.0002; ****, <0.0001).

To get a more complete view of the early host response to infection, we also investigated cytokine and chemokine secretion in the supernatants of BMDM at 24 h postinfection. To do this, we employed the Bio-Plex Pro mouse cytokine 23-plex assay (Bio-Rad). For each condition, secreted protein levels were normalized to those detected in supernatants of BMDM treated with IFN-γ plus LPS for 24 h. We found that cytokine and chemokine secretion was significantly reduced in BMDM infected with both the wild type and the *dotA*::Tn mutant in comparison to that of the IFN-γ plus LPS controls. For both strains of *Coxiella*, there was little secretion of tumor necrosis factor alpha (TNF-α), IL-1β, and IFN-γ (Fig. 5), which have been shown previously to control infection (1, 29, 30). Interestingly, we saw no significant difference in the amounts of IL-3, -5, -6, -9, -13, -17A, or CXCL1 secretion between infected cells and the IFN-γ plus LPS control. Overall, there was little difference in the amount of proinflammatory protein secretion between cells infected with either strain of *Coxiella*, which is consistent with the results of our transcriptional, cytometric, and enzymatic assays (Fig. 3 and 4). However, while overall cytokine and chemokine secretion for infected cells was low, we noted that infection did induce limited secretion in comparison to that of the uninfected mock controls. We concluded that this BMDM model supported NMII replication because the macrophages failed to mount an effective antibacterial response to infection, in contrast to what had been observed in restrictive primary macrophage models of infection (6–8).

### Identification of type IVB secretion effectors that are essential for replication within primary macrophages and SCID mice.

**FIG 6:**
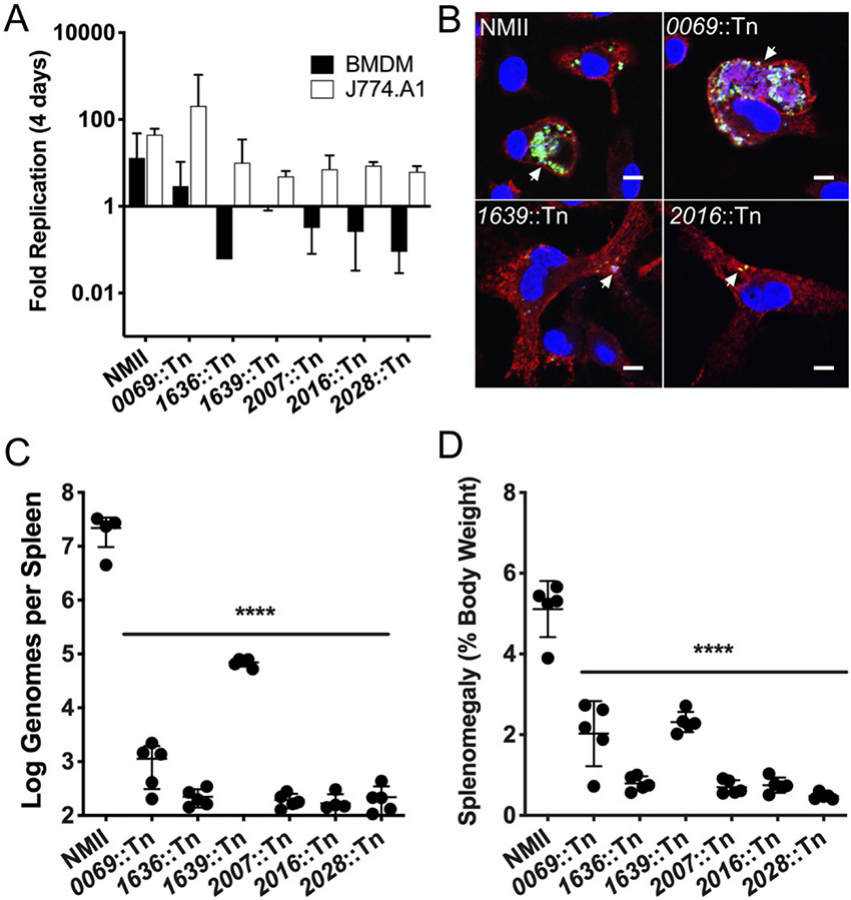
Screen for T4SS effectors required for replication within BMDM. (A) Bar graph showing average fold replication of T4SS effector transposon mutants over 4 days in either BMDM (black) or J774A.1 (white) macrophages. Data shown are the average of results of two independent experiments, with error bars representing standard deviations. (B) Confocal micrographs of representative T4SS effector transposon mutants from the BMDM intracellular replication screen at 4 days postinfection. *Coxiella* was stained green, LAMP-1 is shown in red, and DNA is stained blue with DAPI. Arrowheads indicate the locations of individual CCVs in infected BMDM. Scale bar, 10 μm. (C) Bacterial burden of SCID mouse spleens 14 days postinoculation with wild-type C. burnetii NMII and T4SS effector transposon mutants. (D) Relative spleen weight (splenomegaly) expressed as a percentage of total body weight of mice 14 days postinoculation with wild-type NMII C. burnetii and T4SS effector transposon mutants. For panels C and D, error bars represent standard deviations from the mean (*n* = 5 mice). ****, significance at *P* of <0.0001 by one-way ANOVA for all T4SS mutants tested, relative to results for NMII.

While it appears that the T4SS of C. burnetii is not required for the suppression of host innate immune responses during the earliest stages of infection, it is essential for CCV formation, replication, and innate immune dampening thereafter (Fig. 1) (see references 10 and 11). There are few specific T4SS effectors known to inhibit antibacterial responses to *Coxiella* (12, 13), despite evidence of T4SS-dependent innate immune subversion (31). In fact, genetic disruption of many T4SS effectors resulted in no discernible phenotype during *in vitro* infection of permissive cell lines (14). Having established this BMDM model of C. burnetii NMII infection, we used it to screen for unappreciated T4SS effectors that may contribute to pathogenesis in this cellular context. Among the T4SS transposon mutants in our laboratory (32), we identified five which were capable of replication within the J774.A1 macrophage cell line but that did not replicate within primary murine BMDM (Fig. 6). Replication was completely restricted in BMDM, as in the case of *CBU1639*::Tn, or the bacteria were gradually cleared from the cells (*CBU1636*::Tn, *CBU2007*::Tn, *CBU2016*::Tn, and *CBU2028*::Tn). Interestingly, we did identify a T4SS effector mutant, *CBU0069*::Tn, which replicated within both cell types (Fig. 6). Replication of *CBU0069*::Tn confirms that insertion of the *Himar1* transposon is not sufficient to abrogate *Coxiella* replication; genetic disruption of a specific locus is necessary to observe a replication defect in BMDM. At 4 days postinfection, we saw that the mutants that were cleared by BMDM were limited to tight (nonspacious), LAMP-positive CCVs with individual bacteria, as represented by the *CBU2016*::Tn mutant (Fig. 6B). The *CBU1639*::Tn mutant was consistently found in tight CCVs containing 1 to 3 bacteria. As a result of this screen, we identified three novel virulence phenotypes associated with T4SS effectors of Coxiella burnetii NMII: those that are nonessential for intracellular replication in primary macrophages (*CBU0069*), those that are essential for survival in the context of primary macrophages (*CBU1636*::Tn, *CBU2007*::Tn, *CBU2016*::Tn, and *CBU2028*::Tn), and those that are capable of survival but not replication within BMDM (*CBU1639*::Tn).

To extend these findings, we next assessed the relative virulence of these effector mutants in the SCID mouse model of C. burnetii infection. This mouse model of infection has successfully defined the contribution of virulence loci to *Coxiella* pathogenesis with two readouts of disease severity: bacterial burden in infected tissues and splenomegaly (12, 33). Each of the T4SS mutants that had a BMDM-specific replication defect were used to inoculate SCID mice for 14 days. We observed that each of the transposon mutants was present at a lower density in the spleens of infected mice than were wild-type bacteria, indicating that they are attenuated for virulence (Fig. 5C). While all the T4SS mutants had reduced bacterial burdens, the *CBU1639*::Tn mutant was present in spleen at higher densities than the other T4SS mutants. We were surprised to find that the *CBU0069*::Tn mutant, which replicated efficiently in BMDM, was attenuated in the SCID mouse. The *CBU0069* locus was originally annotated as a T4SS effector (32, 34) and then later reannotated as a pseudogene (14). However, it appears that the *CBU0069* locus encodes some function, as it did contribute to virulence in the SCID mouse. This finding highlights the utility of having multiple assays to evaluate relative virulence, as the contribution of *CBU0069* would have remained unappreciated without the benefit of this animal model.

In addition to measuring bacterial burdens in infected spleens, the weight of each organ was compared to total body weight to measure inflammation due to infection (splenomegaly). Relative to wild-type NMII, each of the T4SS effector mutants induced less splenomegaly, which is consistent with our findings regarding bacterial burdens (Fig. 5D). The mouse infection model confirmed that the T4SS effectors that were required for replication in BMDM do contribute to the virulence of C. burnetii. We also saw that the *CBU1639*::Tn mutant’s phenotype in the animal model was consistent with what we observed *in vitro* and distinct from that of the other mutants we tested. For this reason, we deemed the *CBU1639*::Tn mutant to be of particular interest and worth further investigation.

### *CBU1639*::Tn induces NF-κB-dependent transcription in response to *Coxiella* infection.

**FIG 7:**
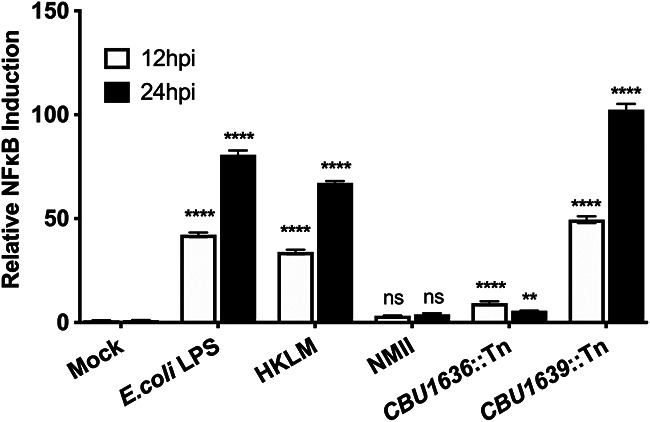
Induction of NF-κB-dependent gene expression by T4SS effector mutant *CBU1639*::Tn. Bar graph illustrating relative fold induction of luciferase by THP1-Lucia reporter cells in response to canonical agonists (E. coli LPS and heat-killed L. monocytogenes [HKLM]) or infection by C. burnetii NMII wild type or T4SS effector mutant strains *CBU1636*::Tn and *CBU1639*::Tn at 12 (white) and 24 (black) hpi. The data presented are the means of results of three independent experiments, and the error bars represent standard deviations from the means. Statistical significance was determined by two-way ANOVA with Dunnett’s test for multiple comparisons. *P* values are reported with asterisks: **, *P* = 0.0010; ****, *P* < 0.0001; ns, not significant.

As the *CBU1639*::Tn mutant had a unique virulence phenotype relative to the other transposon mutants, we subjected it to further study to investigate its contribution to *Coxiella* pathogenesis. We hypothesized that since *CBU1639* is not required in permissive cells for CCV development and intracellular replication, its role may be to contend with the innate immune pathways that are active in primary macrophages but not in permissive cell lines. We used the THP1-Lucia reporter cell line to assess whether the *CBU1639*::Tn strain of *Coxiella* induced proinflammatory gene transcription in response to infection. This human macrophage reporter cell line is exquisitely sensitive to proinflammatory stimuli and produces a wide dynamic range of luciferase in response to NF-κB agonists. We employed this reporter cell line to detect very small differences in the host response to infection with C. burnetii and to distinguish phenotypes between individual transposon mutant strains. THP1 cells that were infected with wild-type NMII express modest amounts of luciferase which were not statistically different from those of the mock-infected controls (Fig. 7). These results were in direct contrast to the significant luciferase expression induced by Escherichia coli LPS and heat-killed Listeria monocytogenes (Fig. 7, HKLM). When these cells were infected with the *CBU1636*::Tn mutant, which is representative of those that are gradually cleared from BMDM (Fig. 7), the induced NF-κB-dependent luciferase expression was modest in comparison to that of the positive controls (up to 80.7-fold for LPS at 24 h postinfection [hpi]). However, the *CBU1639*::Tn mutant induced luciferase production over 100-fold at 24 hpi, demonstrating that this gene may play a role in mitigating the host innate immune response to infection with C. burnetii. Future studies will focus on elucidating the molecular mechanism by which CBU1639 contributes to *Coxiella* pathogenesis in the context of this BMDM model of infection.

## DISCUSSION

As we are interested in understanding the molecular mechanisms of C. burnetii pathogenesis, we sought to adopt a convenient cellular model of infection that more closely represents the host cell environment that it encounters upon infection. Primary macrophages have been successfully applied to the investigation of host responses to *Coxiella* infection (35), but we sought to identify an accessible infection model that did not require the use of human subjects. The comprehensive range of host innate immune pathways and the wide array of genetic tools available in murine BMDM made them a particularly attractive model. We found that differentiation of these cells using conditioned medium from L929 fibroblasts (36) generated host cells that exhibit an unpolarized phenotype prior to stimulation and that respond appropriately to canonical stimuli (Fig. 4) (37, 38). These BMDM permitted intracellular replication of C. burnetii NMII with kinetics comparable to that in other highly permissive cell lines (9, 16, 39). This was very encouraging, as the use of L929-conditioned medium is a convenient and cost-effective method for the differentiation of BMDM *in vitro*.

The transcriptional response to *Coxiella* infection was the first host parameter of this infection model that we queried. We focused on early transcriptional responses to determine how these innate immune cells respond upon initial contact with the bacteria and whether the T4SS contributed to the host cell response prior to bacterial replication. We found that between 8 and 24 hpi, *Coxiella* induced a proinflammatory gene expression program in BMDM that was independent of its T4SS (Fig. 2 and 3). To our knowledge, this is the first report demonstrating a significant induction of host proinflammatory gene transcription earlier than 24 h after inoculation with C. burnetii. At the transcriptional level, C. burnetii-infected BMDM resembled cells stimulated with IFN-γ and LPS, canonical agonists that induce classical activation of macrophages (38). We were surprised to find this, as it contrasted with previous data showing that wild-type NMII inhibits antibacterial host transcription responses via T4SS (11, 12). While previous studies primarily investigated host responses to infection at 24 to 72 h, no significant induction of proinflammatory transcription was observed with wild-type or T4SS-null bacteria prior to 24 h (11, 12). We attribute our ability to detect these early transcriptional responses to the use of primary macrophages, which are more sensitive to invading pathogens than immortalized cell lines (17).

Based on their early differential transcription profile in response to *Coxiella*, we predicted that these BMDM polarized to a classical, proinflammatory phenotype upon infection (Fig. 3 and 4). However, by assessing host cell responses downstream of transcription, such as surface marker expression, enzymatic activity, and protein secretion, we observed no significant upregulation of host defenses (Fig. 4 and 5) that might control bacterial replication. Few studies characterizing the host cell’s response to infection have shown limited induction of antibacterial host responses to C. burnetii NMII in macrophages. In a primary murine alveolar macrophage (AM) model of NMII infection, the infected AMs supported robust intracellular replication, in direct contrast to BMDM that were assessed in parallel (40). Interestingly, these AMs alternatively polarized prior to infection, and that polarization status was sustained after challenge with C. burnetii. In a more recent permissive BMDM infection model using the NMII strain, no polarization of infected BMDM toward classical or an alternative activation state was detected, and modest production of TNF-α and nitrite was observed (9), which is consistent with our findings. Ultimately, we found that the early proinflammatory transcriptional response of BMDM to C. burnetii did not translate into an antibacterial phenotype when downstream markers of macrophage activation were investigated. The observed disconnect between transcription and polarization emphasizes that researchers should exercise caution when interpreting transcriptional responses as indicative of host cell phenotype.

Based on our results with the *dotA*::Tn mutant, if C. burnetii does inhibit early innate immune function prior to replication, it may employ a T4SS-independent strategy. C. burnetii lacks many pathogen-associated molecular patterns (PAMPs) that would alert a professional phagocyte to its presence (2). In addition, lipid A, which is present in both the virulent Nine Mile isolate and the attenuated NMII, has been shown to inactivate TLR4-dependent host responses to infection (41). There is also evidence that C. burnetii can release outer membrane vesicles, which may deliver virulence factors into the host cell across the CCV membrane (42, 43). The sensitivity of the primary BMDM model of infection provides us with an opportunity to investigate such host modulation strategies employed by C. burnetii.

Our permissive BMDM infection model allowed us to identify several T4SS effectors as having a previously unappreciated contribution to *Coxiella* virulence. We screened T4SS effectors that had been described as having no replication defects in HeLa and J774A.1 cells (32) in our BMDM model, hypothesizing that we may find an effector that is required in the context of a primary macrophage among them. We identified five such effectors that were also attenuated in a mouse model of *Coxiella* infection (Fig. 6C and D). These results highlight how our choice of *in vitro* infection model can limit our ability to detect virulence determinants; we must balance convenience with relevance when selecting an experimental platform for discovery.

Among these five effector mutants, we found one, *CBU1639*::Tn, which had a particularly interesting phenotype: in both BMDM and SCID mice, it appeared to survive without replication, in contrast to the other mutants we screened, which were gradually killed and cleared by these hosts. Further investigation with that mutant revealed that it induced antibacterial transcriptional responses upon infection. It is possible that the NF-κB-dependent transcription induced either directly or indirectly by the *CBU1639*::Tn strain was responsible for its growth restriction. We predict that if the function of CBU1639 is required to modulate host responses, it acts later during infection, as our RNA-seq and qRT-PCR data demonstrated that the loss of type IVB secretion does little to affect primary macrophage responses prior to 24 h.

Little is known about the function encoded by the *CBU1639* open reading frame (ORF). The protein was not toxic when expressed ectopically in Saccharomyces cerevisiae, and it did not traffic to a specific cellular compartment when expressed in HeLa cells as a green fluorescent protein (GFP) fusion (32). We employed the I-TASSER protein structure and function prediction server (44) to gain insight into the putative function of this hypothetical protein. I-TASSER returned 10 structural analogs for CBU1639 from the Protein Data Bank (PDB) library (45). Most of those hits aligned CBU1639 with the nucleotide binding adaptor shared by APAF-1, certain *R* gene products, and CED-4 (NB-ARC) domains of proteins involved in the host response to infection, including the apoptosomes of Drosophila melanogaster, Homo sapiens, and Caenorhabditis elegans, as well as Arabidopsis thaliana resistance proteins and the murine NAIP5-NLRC4 inflammasome (see Fig. S1 in the supplemental material). However, CBU1639 was not predicted to include any additional functional domains. While these results do not reveal a definitive function for CBU1639, the structural predictions are consistent with its observed ability to affect innate immune host responses. Future work will focus on confirming the molecular contribution of CBU1639 and other macrophage-specific effectors identified in this work to *Coxiella* pathogenesis and the primary macrophages’ response to infection.

## MATERIALS AND METHODS

### Bacterial strains.

Biosafety level 3- and select agent-exempt C. burnetii RSA439 NMII (clone 4) was used as the parent strain for this study. All mutants used in this study were created using pKM225 containing the *Himar1* transposon (32). C. burnetii wild-type NMII and mutant strains were grown at pH 4.75 in acidified citrate cysteine medium-2 (ACCM-2) from Sunrise Science Products (San Diego, CA, USA) for 7 days at 37°C with 5% CO_2_ and 2.5% O_2_. Cultures were then centrifuged at 15,000 × *g* for 20 min and resuspended in phosphate-buffered saline (PBS), pH 7.4. Genome equivalents for each bacterial stock were determined using quantitative real-time PCR as described below.

### Cell culture and *in vitro* infection assays.

Primary bone marrow-derived macrophages (BMDM) were prepared from C57BL/6 mice (Envigo, Houston, TX) as previously described (15). J774A.1, Vero, and RAW 264.7 cells (all from ATCC) were maintained in Dulbecco modified Eagle medium (DMEM) supplemented with 10% fetal bovine serum (FBS). THP1-Lucia cells (Invivogen) were maintained in RPMI 1640 containing 2 mM l-glutamine, 25 mM HEPES, and 10% heat-inactivated FBS with 200 μg/mL Zeocin (Invivogen) according to the manufacturer’s instructions. All cells were grown at 37°C in 5% CO_2_.

To infect cells with C. burnetii, bacterial stocks from ACCM-2 cultures were enumerated by TaqMan quantitative PCR (qPCR) using primers and a probe directed against the *com1* gene sequence (33) and applied to the cells on ice at a multiplicity of infection (MOI) of 10. The inoculated cells were centrifuged at 500 × *g* for 10 min at 4°C and then shifted to a water bath at 37°C, 5% CO_2_, to synchronize bacterial uptake. After 1 h, the cells were washed 3 times with warm DMEM to remove excess inoculum; the medium was replaced with complete macrophage medium (DMEM supplemented with 10% FBS and 10% L929-conditioned medium) and returned to the incubator for a total of 7 days. The culture medium was replaced daily. To control for infection levels across strains and cell lines, we compared infection efficiencies by fluorescence confocal microscopy (described below) of cells on glass coverslips infected in parallel with those used for downstream analyses. Analysis of infection phenotypes was performed only under conditions where the infection rate was at least 90% for all conditions. For control experiments, 2.5 ng/mL recombinant mouse IFN-γ (R&D Diagnostics) or 20 ng/mL recombinant mouse IL-4 (Peprotech) was added 48 h before infection and maintained throughout the duration of the experiment. IFN-γ-treated BMDM were stimulated with 100 ng/mL E. coli O111:B4 LPS (Invivogen) at infection time zero. For bacterial growth curves, infected cells were collected at 0, 1, 4, and 7 days postinfection (unless indicated otherwise) for genomic DNA isolation and enumeration of bacteria by TaqMan qPCR (33). Three independent infections were performed, and all samples were collected in triplicate.

### RNA-seq and transcriptional analysis.

To prepare RNA samples, BMDM were infected at an MOI of 10 as described above, and RNA was collected from infected cells and mock-infected controls in duplicate at 8 and 24 h postinfection using TRIzol (Invitrogen) according to the manufacturer’s instructions. Total RNA from four independent experiments was quantified with a Qubit flourometer, and quality was assessed on an Agilent TapeStation RNA tape. Stranded mRNA sequencing libraries were generated using the Illumina TruSeq stranded mRNA library prep kit with rRNA depletion and sequenced on an Illumina NextSeq 500 75-cycle high-output sequencing kit.

Raw sequencing reads were checked to trim any adapter sequences and low-quality bases using Trimmomatic (46), after which filtered reads were mapped to the Mus musculus (mm10) genome assembly using version 2.1 (47). Transcript-wise counts were generated using the “featureCounts” tool from the SUBREAD package (48). Differential gene expression tests were then performed using DESeq2 (49) by following recommended guidelines. The resulting gene expression values were entered into Ingenuity Pathway Analysis software (Qiagen, Venlo, Netherlands; www.ingenuity.com) for biological pathway analysis. The complete data set is presented as log-transformed gene expression values in Table S2 in the supplemental material. For prediction of macrophage activation states upon infection, log-transformed gene expression values were input to the MacSpectrum algorithm (28) according to the developers’ instructions. MacSpectrum is freely available at https://macspectrum.uconn.edu.

### Quantitative real-time PCR and RT-PCR.

To enumerate *Coxiella* bacteria, DNA purified from ACCM-2-grown bacteria, infected cells, or infected mouse spleens was used as a template for TaqMan real-time PCR as described previously (33). Twenty-microliter reaction mixtures were run using ABI TaqMan universal PCR master mix on an ABI StepOne Plus machine. For the quantitation of host transcripts, RNA was extracted from 2 × 10^6^ BMDM at 24 h postinfection using TRIzol (Invitrogen) according to the manufacturer’s instructions. Contaminating DNA was removed with a DNA-free DNA removal kit (Invitrogen) according to the manufacturer’s instructions. The purified host transcripts were used as a template for cDNA synthesis with a high-capacity cDNA reverse transcription kit (Invitrogen) using the supplied random primers. Host cDNAs were amplified and quantified with Fast SYBR green master mix (Invitrogen) on an Applied Biosystems QuantStudio 6 machine. A list of the host target transcripts and primers used are included in Table S1. The results are presented as the means of results from three independent experiments. Error bars represent standard deviations from the mean, with asterisks denoting *P* values calculated by a two-tailed ratio-paired *t* test.

### Flow cytometric analysis.

Primary BMDM prepared as described above were seeded into 6-well plates at 3 × 10^5^ cells/well in a 6-well plate 5 days postdifferentiation. After 7 days of differentiation, the BMDM were infected using an MOI of 10 with NMII and NMII *dotA*::Tn or were treated with 2.5 ng/mL recombinant mouse IFN-γ (R&D Diagnostics) or 20 ng/mL recombinant mouse IL-4 (PeproTech) for 48 h. At 48 h, the BMDM were removed from the plates using Accutase (Invitrogen) and enumerated. All samples were placed in 96-well U-bottom plates at a density of 2 × 10^5^ cells/well. The macrophages were first stained for viability using Zombie Red per the manufacturer’s specifications (BioLegend). The cells were then treated with Fc Block 1:100 for 10 min at 4°C (BD Pharmingen). Surface stains (F4/80 biotin [eBioscience] plus phycoerythrin [PE]/Cy7 streptavidin [BioLegend], eFluor450 CD11b [Invitrogen], PE CD11c (Tonbo Bioscience), peridinin chlorophyll protein [PerCP]-Cy5.5 CD38 [BD Pharmingen]) diluted in PBS plus 0.5% bovine serum albumin (PBSA) were incubated with the cells on ice for 20 min. The cells were then washed twice with PBSA and then fixed with 2% paraformaldehyde for 15 min on ice. The cells were then washed once with Perm/Wash (0.1% saponin in PBSA) and permeabilized using Perm/Wash for 20 min on ice. Intracellular stains (Alexa Fluor 488 CD206 [BioLegend] and allophycocyanin [APC] Egr2 [Invitrogen]) diluted in Perm/Wash were incubated with cells on ice for 30 min. Fluorescence-minus-one controls were performed (FMO) with compensation for each fluor using single-stained cells and unstained cells. The stained samples were run on a BD Fortessa analyzer, and analysis was done using FlowJo software.

### Enzymatic activity assays.

The amount of active arginase in 1 × 10^6^ *Coxiella*-infected BMDM was assessed at 24 h postinfection using an arginase activity assay kit (Sigma) according to the manufacturer’s instructions. To detect the presence of active induced nitric oxide synthase (NOS2) in BMDM, the Griess reagent system (Promega) was used to measure the amount of nitrite present at 24 h postinfection in the cell culture supernatants from the same BMDM used for the arginase activity assay. For both assays, duplicate wells were each measured in triplicate, and the results presented are the average of three independent experiments.

### Bio-Plex cytokine assay.

Quantification of secreted cytokines and chemokines was assessed using the Bio-Plex Pro mouse cytokine 23-plex assay (Bio-Rad). BMDM were seeded in a 24-well plate as described above. The cells were infected as described above with wild-type NMII or *dotA*::Tn NMII at an MOI of 10 or were treated with 2.5 ng/mL IFN-γ and 100 ng/mL LPS. Supernatants were collected at 24 h postinfection/treatment in parallel with mock-infected BMDM. Secreted cytokines and chemokines present in the cell culture supernatants were measured using the Bio-Plex Pro mouse cytokine 23-plex assay (Bio-Rad) according to the manufacturer’s instructions. Per the manufacturer’s recommendations, undiluted supernatants were measured in triplicate and quantified using a five-parameter logistic regression with an 8-sample standard curve. Results were calculated as the average of triplicate samples in pg/mL. The mean protein secretion from three independent experiments for each condition was normalized to that of the positive control (IFN-γ plus LPS). Data for secretion of MIP-1β is not reported, as it was outside the limit of detection for the positive control. Error bars represent standard deviations from the mean, with asterisks denoting *P* values calculated by two-way analysis of variance (ANOVA), with Dunnett’s test for multiple comparisons.

### Western blotting.

At 24 h postinfection, BMDM were lysed directly in a 6-well tissue culture dish with hot 2× NuPAGE LDS sample buffer (Invitrogen) and boiled for 10 min. The resultant whole-cell lysates were loaded onto a 4-to-12% SDS-PAGE gel for electrophoresis and transferred to a polyvinylidene difluoride (PVDF) membrane for probing. The following antibodies were used for primary probing: rabbit anti-NOS2, rabbit anti-CD38, rabbit anti-ARG1 (all from AbClonal). Mouse anti-actin (Sigma) was used as a loading control for the assay. For detection, we used LI-COR IRDye 800CW donkey anti-rabbit and IRDye 680RD donkey anti-mouse secondary antibodies and imaged blots with a LI-COR Odyssey Fc imager (Lincoln, NE).

### SCID mouse infections.

SCID (C.B-17/lcrHsd-*Prkdc*^scid^) mice were purchased from Envigo (Indianapolis, IN, USA) and housed in the Texas A&M University Health Science Center (TAMHSC) animal facility. All animal procedures were done in compliance with Texas A&M University IACUC (AUP no. 2016-0370). Infections were performed as described previously (33). Briefly, 6- to 8-week-old female mice (SCID or C57BL/6) were infected with 1 × 10^6^ C. burnetii phase II strain bacteria via intraperitoneal (i.p.) injection. DNA was extracted from spleens at 14 days postinfection as previously described (12) for bacterial enumeration.

### Fluorescence microscopy.

Cells seeded on 12-mm glass coverslips were washed 3 times with PBS and then fixed with room-temperature 4% paraformaldehyde at the indicted times postinfection. After fixation, the coverslips were again washed with PBS and stored at 4°C prior to immunofluorescence staining. Staining proceeded as described previously (15), using guinea pig NMII antisera (J. Samuel Laboratory, TAMHSC) to detect *Coxiella*, rat anti-LAMP1 antibody (1D4B; Developmental Studies Hybridoma Bank), and 10 ng/mL DAPI (4′,6-diamidino-2-phenylindole). Images were captured with a Nikon A1 confocal microscope and processed using Nikon Elements software and Adobe Photoshop.

### THP1-Lucia luciferase assay.

A total of 1 × 10^6^ THP1-Lucia cells were stimulated with heat-killed Listeria monocytogenes (10^7^ cells/mL) or *E. coli* LPS O111:B4 (200 ng/mL) or infected at an MOI of 25 with either wild-type C. burnetii NMII, C. burnetii NMII *CBU1639*::Tn, or C. burnetii NMII *CBU1636*::Tn. At 12 and 24 h postinfection, the cell culture supernatants were collected, and the levels of NF-κB-induced luminescence were measured in triplicate from the cell culture supernatant using QUANTI-Luc coelenterazine-based luminescence assay detection reagent (Invivogen) on a PerkinElmer EnVision 2104 multilabel reader. A total of three independent experiments were performed, and the data were normalized to those of the mock-treated control to generate NF-κB fold induction.

### Data availability.

The RNA-seq data set has been deposited in the GEO database under accession number GSE208339.

## References

[1] Graham JG, MacDonald LJ, Hussain SK, Sharma UM, Kurten RC, Voth DE. 2013. Virulent Coxiella burnetii pathotypes productively infect primary human alveolar macrophages. Cell Microbiol 15:1012–1025. doi:10.1111/cmi.12096. 23279051 PMC3655087

[2] van Schaik EJ, Chen C, Mertens K, Weber MM, Samuel JE. 2013. Molecular pathogenesis of the obligate intracellular bacterium Coxiella burnetii. Nat Rev Microbiol 11:561–573. doi:10.1038/nrmicro3049. 23797173 PMC4134018

[3] Brooke RJ, Mutters NT, Peter O, Kretzschmar ME, Teunis PF. 2015. Exposure to low doses of Coxiella burnetii caused high illness attack rates: insights from combining human challenge and outbreak data. Epidemics 11:1–6. doi:10.1016/j.epidem.2014.12.004. 25979276

[4] Scott GH, Williams JC. 1990. Susceptibility of Coxiella burnetii to chemical disinfectants. Ann N Y Acad Sci 590:291–296. doi:10.1111/j.1749-6632.1990.tb42235.x. 2378460

[5] Williams JC, Peacock MG, McCaul TF. 1981. Immunological and biological characterization of Coxiella burnetii, phases I and II, separated from host components. Infect Immun 32:840–851. doi:10.1128/iai.32.2.840-851.1981. 7251150 PMC351520

[6] Zamboni DS, Rabinovitch M. 2003. Nitric oxide partially controls Coxiella burnetii phase II infection in mouse primary macrophages. Infect Immun 71:1225–1233. doi:10.1128/IAI.71.3.1225-1233.2003. 12595436 PMC148841

[7] Bradley WP, Boyer MA, Nguyen HT, Birdwell LD, Yu J, Ribeiro JM, Weiss SR, Zamboni DS, Roy CR, Shin S. 2016. Primary role for Toll-like receptor-driven tumor necrosis factor rather than cytosolic immune detection in restricting Coxiella burnetii phase II replication within mouse macrophages. Infect Immun 84:998–1015. doi:10.1128/IAI.01536-15. 26787725 PMC4807492

[8] Zamboni DS. 2004. Genetic control of natural resistance of mouse macrophages to Coxiella burnetii infection in vitro: macrophages from restrictive strains control parasitophorous vacuole maturation. Infect Immun 72:2395–2399. doi:10.1128/IAI.72.4.2395-2399.2004. 15039367 PMC375151

[9] Cockrell DC, Long CM, Robertson SJ, Shannon JG, Miller HE, Myers L, Larson CL, Starr T, Beare PA, Heinzen RA. 2017. Robust growth of avirulent phase II Coxiella burnetii in bone marrow-derived murine macrophages. PLoS One 12:e0173528. doi:10.1371/journal.pone.0173528. 28278296 PMC5344453

[10] Beare PA, Larson CL, Gilk SD, Heinzen RA. 2012. Two systems for targeted gene deletion in Coxiella burnetii. Appl Environ Microbiol 78:4580–4589. doi:10.1128/AEM.00881-12. 22522687 PMC3370473

[11] Clemente TM, Mulye M, Justis AV, Nallandhighal S, Tran TM, Gilk SD. 2018. Coxiella burnetii blocks intracellular interleukin-17 signaling in macrophages. Infect Immun 86:e00532-18. doi:10.1128/IAI.00532-18. 30061378 PMC6204741

[12] Burette M, Allombert J, Lambou K, Maarifi G, Nisole S, Di Russo Case E, Blanchet FP, Hassen-Khodja C, Cabantous S, Samuel J, Martinez E, Bonazzi M. 2020. Modulation of innate immune signaling by a Coxiella burnetii eukaryotic-like effector protein. Proc Natl Acad Sci USA 117:13708–13718. doi:10.1073/pnas.1914892117. 32482853 PMC7306807

[13] Cunha LD, Ribeiro JM, Fernandes TD, Massis LM, Khoo CA, Moffatt JH, Newton HJ, Roy CR, Zamboni DS. 2015. Inhibition of inflammasome activation by Coxiella burnetii type IVB secretion system effector IcaA. Nat Commun 6:10205. doi:10.1038/ncomms10205. 26687278 PMC4703858

[14] Larson CL, Martinez E, Beare PA, Jeffrey B, Heinzen RA, Bonazzi M. 2016. Right on Q: genetics begin to unravel Coxiella burnetii host cell interactions. Future Microbiol 11:919–939. doi:10.2217/fmb-2016-0044. 27418426 PMC5619019

[15] Chong A, Wehrly TD, Nair V, Fischer ER, Barker JR, Klose KE, Celli J. 2008. The early phagosomal stage of Francisella tularensis determines optimal phagosomal escape and Francisella pathogenicity island protein expression. Infect Immun 76:5488–5499. doi:10.1128/IAI.00682-08. 18852245 PMC2583578

[16] Zamboni DS, Mortara RA, Rabinovitch M. 2001. Infection of Vero cells with Coxiella burnetii phase II: relative intracellular bacterial load and distribution estimated by confocal laser scanning microscopy and morphometry. J Microbiol Methods 43:223–232. doi:10.1016/s0167-7012(00)00223-2. 11118656

[17] Andreu N, Phelan J, de Sessions PF, Cliff JM, Clark TG, Hibberd ML. 2017. Primary macrophages and J774 cells respond differently to infection with Mycobacterium tuberculosis. Sci Rep 7:42225. doi:10.1038/srep42225. 28176867 PMC5296737

[18] Medzhitov R, Horng T. 2009. Transcriptional control of the inflammatory response. Nat Rev Immunol 9:692–703. doi:10.1038/nri2634. 19859064

[19] Newton HJ, McDonough JA, Roy CR. 2013. Effector protein translocation by the Coxiella burnetii Dot/Icm type IVB secretion system requires endocytic maturation of the pathogen-occupied vacuole. PLoS One 8:e54566. doi:10.1371/journal.pone.0054566. 23349930 PMC3547880

[20] Heap RE, Marin-Rubio JL, Peltier J, Heunis T, Dannoura A, Moore A, Trost M. 2021. Proteomics characterisation of the L929 cell supernatant and its role in BMDM differentiation. Life Sci Alliance 4:e202000957. doi:10.26508/lsa.202000957. 33853969 PMC8091624

[21] Myers MJ, Pullen JK, Ghildyal N, Eustis-Turf E, Schook LB. 1989. Regulation of IL-1 and TNF-alpha expression during the differentiation of bone marrow derived macrophage. J Immunol 142:153–160. 2642503

[22] Pullen JK, Eustis-Turf E, Myers MJ, Schook LB. 1989. Regulation of MHC gene expression during the differentiation of bone marrow-derived macrophages. Cell Immunol 121:398–413. doi:10.1016/0008-8749(89)90039-7. 2500255

[23] Schook LB, Gutmann DH, Marlin LE, Niederhuber JE. 1984. In-vitro-derived bone marrow macrophages. Expression of Ia antigens during macrophage differentiation. Transplantation 37:585–590. doi:10.1097/00007890-198406000-00012. 6427997

[24] Ornatowska M, Azim AC, Wang X, Christman JW, Xiao L, Joo M, Sadikot RT. 2007. Functional genomics of silencing TREM-1 on TLR4 signaling in macrophages. Am J Physiol Lung Cell Mol Physiol 293:L1377–L1384. doi:10.1152/ajplung.00140.2007. 17905855 PMC3969455

[25] Pan W, Yu C, Hsuchou H, Kastin AJ. 2010. The role of cerebral vascular NFkappaB in LPS-induced inflammation: differential regulation of efflux transporter and transporting cytokine receptors. Cell Physiol Biochem 25:623–630. doi:10.1159/000315081. 20511707 PMC2910582

[26] Mehraj V, Textoris J, Ben Amara A, Ghigo E, Raoult D, Capo C, Mege JL. 2013. Monocyte responses in the context of Q fever: from a static polarized model to a kinetic model of activation. J Infect Dis 208:942–951. doi:10.1093/infdis/jit266. 23801603

[27] Castrillo A, Joseph SB, Vaidya SA, Haberland M, Fogelman AM, Cheng G, Tontonoz P. 2003. Crosstalk between LXR and toll-like receptor signaling mediates bacterial and viral antagonism of cholesterol metabolism. Mol Cell 12:805–816. doi:10.1016/s1097-2765(03)00384-8. 14580333

[28] Li C, Menoret A, Farragher C, Ouyang Z, Bonin C, Holvoet P, Vella AT, Zhou B. 2019. Single cell transcriptomics based-MacSpectrum reveals novel macrophage activation signatures in diseases. JCI Insight 5:e126453. doi:10.1172/jci.insight.126453. 30990466 PMC6542613

[29] Zamboni DS, Campos MA, Torrecilhas AC, Kiss K, Samuel JE, Golenbock DT, Lauw FN, Roy CR, Almeida IC, Gazzinelli RT. 2004. Stimulation of toll-like receptor 2 by Coxiella burnetii is required for macrophage production of pro-inflammatory cytokines and resistance to infection. J Biol Chem 279:54405–54415. doi:10.1074/jbc.M410340200. 15485838

[30] Hedges JF, Robison A, Kimmel E, Christensen K, Lucas E, Ramstead A, Jutila MA. 2016. Type I interferon counters or promotes Coxiella burnetii replication dependent on tissue. Infect Immun 84:1815–1825. doi:10.1128/IAI.01540-15. 27068091 PMC4907146

[31] Mahapatra S, Gallaher B, Smith SC, Graham JG, Voth DE, Shaw EI. 2016. Coxiella burnetii employs the Dot/Icm type IVB secretion system to modulate host NF-kappaB/RelA activation. Front Cell Infect Microbiol 6:188. doi:10.3389/fcimb.2016.00188. 28066723 PMC5165255

[32] Weber MM, Chen C, Rowin K, Mertens K, Galvan G, Zhi H, Dealing CM, Roman VA, Banga S, Tan Y, Luo Z-Q, Samuel JE. 2013. Identification of Coxiella burnetii type IVB secretion substrates required for intracellular replication and Coxiella-containing vacuole formation. J Bacteriol 195:3914–3924. doi:10.1128/JB.00071-13. 23813730 PMC3754607

[33] van Schaik EJ, Case ED, Martinez E, Bonazzi M, Samuel JE. 2017. The SCID mouse model for identifying virulence determinants in Coxiella burnetii. Front Cell Infect Microbiol 7:25. doi:10.3389/fcimb.2017.00025. 28217558 PMC5289997

[34] Seshadri R, Paulsen IT, Eisen JA, Read TD, Nelson KE, Nelson WC, Ward NL, Tettelin H, Davidsen TM, Beanan MJ, Deboy RT, Daugherty SC, Brinkac LM, Madupu R, Dodson RJ, Khouri HM, Lee KH, Carty HA, Scanlan D, Heinzen RA, Thompson HA, Samuel JE, Fraser CM, Heidelberg JF. 2003. Complete genome sequence of the Q-fever pathogen Coxiella burnetii. Proc Natl Acad Sci USA 100:5455–5460. doi:10.1073/pnas.0931379100. 12704232 PMC154366

[35] Dragan AL, Kurten RC, Voth DE. 2019. Characterization of early stages of human alveolar infection by the Q fever agent Coxiella burnetii. Infect Immun 87:e00028-19. doi:10.1128/IAI.00028-19. 30833339 PMC6479048

[36] Yamamoto-Yamaguchi Y, Tomida M, Hozumi M. 1983. Effect of mouse interferon on growth and differentiation of mouse bone marrow cells stimulated by two different types of colony-stimulating factor. Blood 62:597–601. doi:10.1182/blood.V62.3.597.597. 6603878

[37] Orecchioni M, Ghosheh Y, Pramod AB, Ley K. 2019. Macrophage polarization: different gene signatures in M1(LPS+) vs. classically and M2(LPS−) vs. alternatively activated macrophages. Front Immunol 10:1084. doi:10.3389/fimmu.2019.01084. 31178859 PMC6543837

[38] Huang X, Li Y, Fu M, Xin HB. 2018. Polarizing macrophages in vitro. Methods Mol Biol 1784:119–126. doi:10.1007/978-1-4939-7837-3_12. 29761394 PMC8875934

[39] Sanchez SE, Goodman AG, Omsland A. 2021. Metabolic plasticity aids amphotropism of Coxiella burnetii. Infect Immun 89:e00135-21. doi:10.1128/IAI.00135-21. 34491791 PMC8594591

[40] Fernandes TD, Cunha LD, Ribeiro JM, Massis LM, Lima-Junior DS, Newton HJ, Zamboni DS. 2016. Murine alveolar macrophages are highly susceptible to replication of Coxiella burnetii phase II in vitro. Infect Immun 84:2439–2448. doi:10.1128/IAI.00411-16. 27297388 PMC4995897

[41] Conti F, Boucherit N, Baldassarre V, Trouplin V, Toman R, Mottola G, Mege JL, Ghigo E. 2014. Coxiella burnetii lipopolysaccharide blocks p38alpha-MAPK activation through the disruption of TLR-2 and TLR-4 association. Front Cell Infect Microbiol 4:182. doi:10.3389/fcimb.2014.00182. 25610812 PMC4285172

[42] Stead CM, Omsland A, Beare PA, Sandoz KM, Heinzen RA. 2013. Sec-mediated secretion by Coxiella burnetii. BMC Microbiol 13:222. doi:10.1186/1471-2180-13-222. 24093460 PMC3882888

[43] Sandoz KM, Moore RA, Beare PA, Patel AV, Smith RE, Bern M, Hwang H, Cooper CJ, Priola SA, Parks JM, Gumbart JC, Mesnage S, Heinzen RA. 2021. beta-Barrel proteins tether the outer membrane in many Gram-negative bacteria. Nat Microbiol 6:19–26. doi:10.1038/s41564-020-00798-4. 33139883 PMC7755725

[44] Yang J, Zhang Y. 2015. I-TASSER server: new development for protein structure and function predictions. Nucleic Acids Res 43:W174–W181. doi:10.1093/nar/gkv342. 25883148 PMC4489253

[45] Berman HM, Westbrook J, Feng Z, Gilliland G, Bhat TN, Weissig H, Shindyalov IN, Bourne PE. 2000. The Protein Data Bank. Nucleic Acids Res 28:235–242. doi:10.1093/nar/28.1.235. 10592235 PMC102472

[46] Bolger AM, Lohse M, Usadel B. 2014. Trimmomatic: a flexible trimmer for Illumina sequence data. Bioinformatics 30:2114–2120. doi:10.1093/bioinformatics/btu170. 24695404 PMC4103590

[47] Kim D, Langmead B, Salzberg SL. 2015. HISAT: a fast spliced aligner with low memory requirements. Nat Methods 12:357–360. doi:10.1038/nmeth.3317. 25751142 PMC4655817

[48] Liao Y, Smyth GK, Shi W. 2014. featureCounts: an efficient general purpose program for assigning sequence reads to genomic features. Bioinformatics 30:923–930. doi:10.1093/bioinformatics/btt656. 24227677

[49] Love MI, Huber W, Anders S. 2014. Moderated estimation of fold change and dispersion for RNA-seq data with DESeq2. Genome Biol 15:550. doi:10.1186/s13059-014-0550-8. 25516281 PMC4302049

