# Supplementary material for: Primary Murine Macrophages as a Tool for Virulence Factor Discovery in Coxiella burnetii
Source: Microbiol Spectr. 2022 Aug 1;10(4):e02484-21. doi: 10.1128/spectrum.02484-21 (PMC9430109; doi:10.1128/spectrum.02484-21)
Supplement: Supplemental file 1 — Fig. S1. Download spectrum.02484-21-s0001.pdf, PDF file, 0.4 MB [file spectrum.02484-21-s0001.pdf]

A

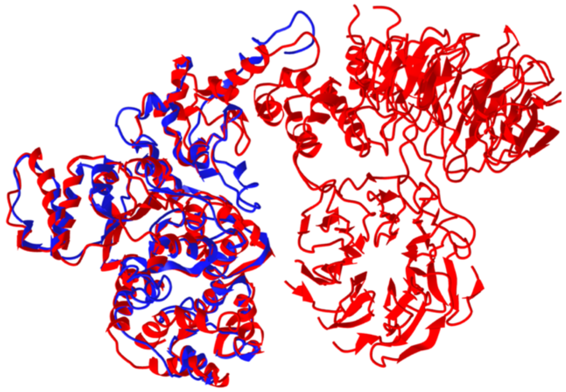

B

Rank	PDB Hit	Structural Analog	TM-Score	RMSD[a]	IDEN[b]	Cov
**1**	1vt4I	*D. melanogaster* apoptosome	0.828	1.98	0.149	0.877
**2**	5julA	*D. melangaster* Dark apoptosome	0.764	2.97	0.138	0.872
**3**	3izaA	*H. sapiens* apoptosome	0.598	3.83	0.079	0.731
**4**	3lqqA	*C. elegans* CED-4 apoptosome	0.589	3.64	0.081	0.704
**5**	6j5tG	*A. thaliana* NLR resistosome	0.558	3.81	0.085	0.681
**6**	7crcA	*A. thaliana* NLR RPP1 complex	0.549	4.15	0.101	0.683
**7**	6b5bA	*M. musculus* NAIP5-NLRC4 inflammasome	0.533	6.09	0.094	0.791
**8**	7jlvA	*N. benthamiana* Roq1 resistosome	0.528	3.86	0.100	0.653
**9**	2fnaA	*S. sulfataricus* AAA+ ATPase	0.503	3.66	0.094	0.608
**10**	2qenA	*P. abyssi* Walker-Type ATPase	0.498	3.70	0.083	0.598

Figure S1. I-TASSER Protein Folding Predictions for CBU1639. The amino acid sequence of CBU1639 was submitted to the I-TASSER server (available at https://zhanggroup.org/I-TASSER/) for tertiary structure and function prediction (43). (A) Ribbon diagram of the top PDB threading template returned by I-TASSER (1vt4I, red) overlaid with the highest-confidence I-TASSER-generated structure for CBU1639 (blue). (B) Table summarizing the top ten identified structural analogs for CBU1639 from PDB (https://www.rcsb.org). Ranking of proteins is based on TM-score of the structural alignment between the query structure and known structures in the PDB library. RMSD[a] is the root-mean-square deviation in distance between residues that are structurally aligned by TM-align. IDEN[b] is the percentage sequence identity in the structurally aligned region. Cov represents the coverage of the alignment by TM-align and is equal to the number of structurally aligned residues divided by length of the query protein.
