# Supplementary material for: Primary Murine Macrophages as a Tool for Virulence Factor Discovery in Coxiella burnetii
Source: Microbiol Spectr. 2022 Aug 1;10(4):e02484-21. doi: 10.1128/spectrum.02484-21 (PMC9430109; doi:10.1128/spectrum.02484-21)
Supplement: Supplemental file 2 — Table S1. Download spectrum.02484-21-s0002.pdf, PDF file, 0.03 MB [file spectrum.02484-21-s0002.pdf]

Table S1. Oligonucleotides used in this Study

Sequence (5' to 3')	Description	Reference
GCGTTGTCTTCAAAGAACTGCC	*com1* qPCR forward	This study
GCGCGTCGTGGAAAGCATAA	*com1* qPCR reverse	This study
FAM-CGGCCAATCGCAATACGCTG-TAMRA	*com1* qPCR probe	(1)
CAGTATGGCTACCTACCTCCAG	*mmp14* qRT-PCR forward	(2)
GCCTTGCCTGTCACTTGTAAA	*mmp14* qRT-PCR reverse	(2)
GAGCCTGGCTAGGAAGGTG	*fpr2* qRT-PCR forward	(2)
TGCTGAAACCAATAAGGAACCTG	*fpr2* qRT-PCR reverse	(2)
TCTCTAGGAAAGCCCAGATCG	*cd38* qRT-PCR forward	(2)
AGAAAAGTGCTTCGTGGTAGG	*cd38* qRT-PCR reverse	(2)
GCTGGCCTGCCTAAAAGATACTG	*saa3* qRT-PCR forward	(3)
GCATTTCACAAGTATTTATTCAGC	*saa3* qRT-PCR reverse	(3)
AATCCATTTGCTGCCTTCCG	*rasgrp1* qRT-PCR forward	This study
TGCAGGCATGTCAAAGTCAC	*rasgrp1* qRT-PCR reverse	This study
GGCCTCAAGGACGACAACA	*lcn2* qRT-PCR forward	(4)
GCATCCCAGTCAGCCACACT	*lcn2* qRT-PCR reverse	(4)

References:

1. van Schaik EJ, Case ED, Martinez E, Bonazzi M, Samuel JE. 2017. The SCID mouse model for identifying virulence determinants in Coxiella burnetii. Front Cell Infect Microbiol 7:25. https://doi.org/10.3389/fcimb.2017.00025.

2. Wang X, Spandidos A, Wang H, Seed B. 2012. PrimerBank: a PCR primer database for quantitative gene expression analysis, 2012 update. Nucleic Acids Res 40:D1144–D1149. https://doi.org/10.1093/nar/gkr1013.

3. Eckhardt ER, Witta J, Zhong J, Arsenescu R, Arsenescu V, Wang Y, Ghoshal S, de Beer MC, de Beer FC, de Villiers WJ. 2010. Intestinal epithelial serum amyloid A modulates bacterial growth in vitro and pro-inflammatory responses in mouse experimental colitis. BMC Gastroenterol 10:133. https://doi.org/10.1186/1471-230X-10-133.

4. Nelson AL, Barasch JM, Bunte RM, Weiser JN. 2005. Bacterial colonization of nasal mucosa induces expression of siderocalin, an iron-sequestering component of innate immunity. Cell Microbiol 7:1404–1417. https://doi.org/10.1111/j.1462-5822.2005.00566.x.
